# The ethical aspects of human organ-on-chip models: A mapping review

**DOI:** 10.1016/j.stemcr.2025.102686

**Published:** 2025-10-30

**Authors:** Jesse Weidema, Martine de Vries, Christine Mummery, Nienke de Graeff

**Affiliations:** 1 Medical Ethics and Health Law, Leiden University Medical Center, Leiden, the Netherlands; 2 Anatomy and Embryology, Leiden University Medical Center, Leiden, the Netherlands

**Keywords:** organ-on-chips, organoids, personalized medicine, precision medicine, digital twins, ethics, informed consent, stem cell research

## Abstract

Organ-on-chips (OoCs) are controlled microfluidic platforms that replicate specific organ-level functionalities and pathological processes using cultured cells. OoCs promise to enhance drug discovery, reduce dependence on animal models, and enable personalized treatments. However, OoCs also introduce ethical challenges. This article provides a mapping review of the philosophical and ethical issues associated with developing and using OoCs. Given the limited literature on OoC ethics, the review takes a comparative analytical approach, drawing on organoid, digital twin, and precision medicine literature. Ethical issues are categorized across three consecutive phases—research, clinical testing, and implementation. Nine key themes are identified: privacy and confidentiality, informed consent, evidence, ontology and moral status, animal experimentation, evidence standards, patient care, intellectual property and commercialization, and distributive justice. Overall, the review highlights several key challenges that require further normative inquiry and hold significance for both research and policy. These include underrepresented groups in OoC research, complexities and limitations related to different consent models, the need for clear criteria to determine evidence standards for replacing animal models, accountability in the standardization of OoC research, and sustainability.

## Teaser

In this article, Weidema and colleagues map philosophical and ethical issues in organ-on-chip research. Drawing on organoid, digital twin, and precision medicine literatures, they identify nine themes across research, testing, and implementation. The review highlights several challenges in need of further normative inquiry, including underrepresented groups, consent models, evidence standards, accountability in standardization, and sustainability.

## Introduction

Organ-on-chips (OoCs) are controlled microfluidic platforms engineered to contain (human) cells that emulate specific organ-level functionalities and pathological processes (Ingber, 2022; Low et al., 2021). Some examples include a “breathing” lung-on-a-chip, which reproduces organ-level physiological and pathophysiological responses to bacteria and (airborne) environmental toxins in the alveolar space (Huh et al., 2010); a gut-on-a-chip, which emulates the intestine’s villus structure that enables the study of intestinal pathophysiology, host-microbiome interactions, and drug absorption processes (Kim et al., 2015); and a fluidically coupled multi-organ chip system used to study metastatic tumor progression throughout different organs (Skardal et al., 2016). Additionally, OoC research extends to modeling various other critical organs and systems, including the heart (Kujala et al., 2016), liver (Sarkar et al., 2015), retina (Chung et al., 2018), bone, and brain (Pediaditakis et al., 2021), as well as the lymphoid and reproductive systems (Blundell et al., 2017).

These OoC models—also called microphysiological systems (MPSs) or tissue chips (Low et al., 2021)—offer several advantages for functional testing compared to traditional culture platforms because they allow for higher levels of control over the “physico-chemical” parameters of cells and tissues within a single culture system (van den Berg et al., 2019). By allowing close control of factors such as oxygen levels, nutrient flow, and organ-specific mechanical forces like breathing and shear stress, as well as the integrated circulation of bacteria and immune cells, tissue chips can faithfully recapitulate certain biological conditions found within the human body (Ingber, 2022). This high level of control can facilitate relevant physiological responses in studies of drug toxicity screening, disease modeling, and drug target discovery and development. Moreover, tissue chips promise to contribute to the 3Rs for replacing, reducing, and refining the use of animal testing models in research by providing an animal-free alternative (Ingber, 2020). Additionally, an important opportunity emerges when patient-derived primary cells, organoids, or induced pluripotent stem cell (iPSC) derivatives are integrated within tissue chip systems. These could then be leveraged to develop or select personalized treatments for individual patients or subpopulations with specific disease comorbidities, potentially transforming the design of clinical trials (Ingber, 2022; Low et al., 2021; van den Berg et al., 2019).

While tissue chips offer significant potential to advance biomedical and fundamental research, they also raise ethical issues. Tissue chips utilize human biological material, produce sensitive personal data, may involve long-term biobank storage, and could potentially create semi-biological entities capable of resembling elements of cognition or consciousness. These ethical issues must be properly addressed to ensure the responsible innovation and application of tissue chip technology in science, medicine, and healthcare. However, although the scientific literature widely discusses the rapid expansion and technological advancements of tissue chip research, and the technology is increasingly reflected in relevant regulations (Box 1), its ethical implications remain largely unexplored.

Box 1 **Regulatory developments relevant to tissue chip and organoid research**

Recent policy changes in the United States and Europe illustrate a gradual but significant regulatory receptivity toward non-animal approaches in drug development, particularly organoids and tissue chip systems. These shifts reflect both practical interest in alternative testing methods and the growing infrastructural alignment required for integrating such methods into regulatory frameworks.

The U.S. Food and Drug Administration's (FDA) *Modernization Act 2.0* has removed statutory requirements for animal testing in certain drug development contexts, explicitly allowing New Approach Methods (NAMs), including organoids and OoCs, to be used in investigational drug applications (Han, 2023). Similarly, the FDA and National Institutes of Health (NIH) have released a roadmap (U.S. Food and Drug Administration, 2025) to support the validation, standardization, and adoption of these systems in regulatory frameworks. The U.S. Government Accountability Office (GAO) has also assessed OoC technologies, identifying their potential benefits, challenges for development, use, and policy options to address these challenges (U.S. Government Accountability Office, 2025). In Europe, the Joint Research Centre identified the need for standardization in tissue chip research and contributed to the formation of the European Committee for Electrotechnical Standardization Organ-on-Chip Focus Group (CEN/CENELEC FGOoC). Together with the European Organ-on-Chip Society (EUROoCS) and the Royal Netherlands Standardization Institute (NEN), this group has published a roadmap (CEN/CENELEC, 2024) that sets priorities for harmonization and the integration of these technologies into regulatory frameworks.

To support responsible scientific and regulatory development, this study presents a mapping review of the philosophical and ethical dimensions of tissue chip technologies. Because the dedicated ethics literature on tissue chips is limited, the review takes a comparative analytical approach, drawing on the organoid, precision medicine (PM), and digital twin (DT) literature (see methods). This approach enables the identification of areas that are well addressed and underexplored within the sampled literature, highlights themes that warrant further normative inquiry or attention in governance frameworks, and offers policy recommendations where appropriate.

## Results

**Figure 1:**
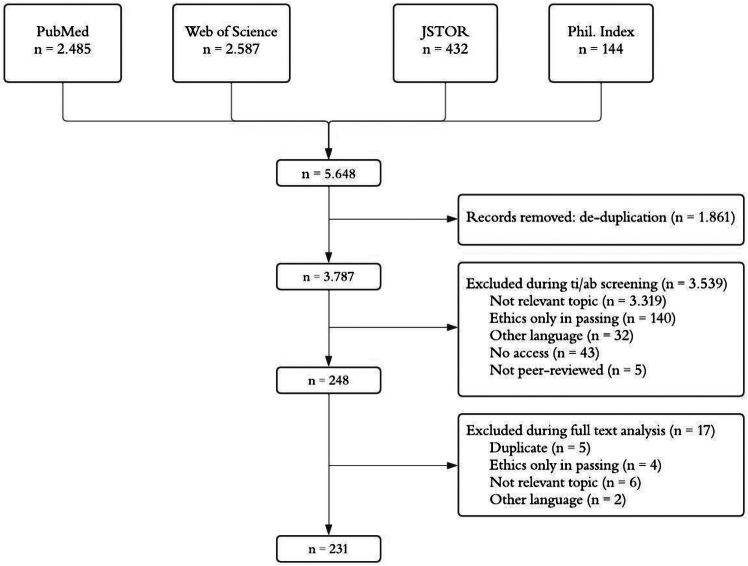
Flow diagram of the included literature Database searches (April 2024 and January 2025) identified 5,648 records. After de-duplication (1,861 records removed), 3,787 records remained. Following title/abstract screening and full-text analysis, 231 articles were included: 5 on tissue chips, 80 on organoids, 127 on PM, and 19 on DTs.

A first database search was conducted on April 24, 2024, identifying 4,873 unique articles. A second search was conducted on January 10, 2025, targeting the period between April 24, 2024, and January 10, 2025, which resulted in 775 additional articles. This resulted in a combined total of 5,648 records. After deduplication and title/abstract screening, 231 articles were included for review: 5 on tissue chips, 80 on organoids, 127 on PM, and 19 on DTs (Figure 1).

To further contextualize the ethical considerations discussed in the mapped literature, we developed a phase-specific ethical map (Table 1) that categorizes the identified ethical themes into three broad phases: research, clinical testing, and implementation (cf. van Daal et al., 2023). Although some ethical themes cut across multiple phases, we discuss each within the phase where it is most applicable, based on consensus among the authors. Furthermore, we map the identified themes onto bioethical principles such as respect for autonomy, beneficence, non-maleficence, and justice where relevant to clarify their ethical significance within established biomedical practice.

**Table 1:** Phase-specific ethical map

Phase	Sub-theme	Ethical issue	Explanation
Research	Privacy and confidentiality	Unauthorized access and re-identification of anonymized data	Using sensitive biobank data in tissue chip research increases the risk of re-identification and misuse by third parties. Unauthorized access could result in material and immaterial harm.
	Informed consent	Autonomy and uncertainty	The scope of data collection is often unclear. Meaningful consent is, therefore, difficult to establish.
	Evidence	Evidence can be inconclusive, misguided or inscrutable	Tissue chips may generate probable yet uncertain results, suffer from input biases that limit applicability across diverse populations, or produce predictions that are difficult to interpret and reproduce.
	Ontology and moral status	Moral status, hybrid status, hype and false hope	Concerns about models developing human-like attributes provoke ethical considerations similar to those for sentient animals. Hybrid status can provoke personal attachment and influence donor perception. Media-driven hype could foster unrealistic expectations and public mistrust.
	Animal experimentation	Animal welfare and the justification for continued use of animal models	Despite advances in tissue chip technology, animal models are still widely used, raising concerns about their well-being. The “3Rs” aim to reduce animal suffering and harm. Integrating tissue chips requires conclusive data and interdisciplinary collaboration.
Clinical testing	Evidence standards in translational research	First-in-human trials, regulatory ambiguity, overdiagnosis	Tissue chip-based preclinical data may not translate to human outcomes, raising questions about safety. The dual-purpose nature of tissue chips blurs boundaries between research and clinical care. Overdiagnosis can occur when tissue chips detect low-risk biomarkers, leading to unnecessary medical interventions.
	Patient care	Liability, resource allocation, incidental findings	Integrating tissue chips may increase errors and liability risks, burden healthcare systems with training demands, and reduce patient interaction time. Incidental findings raise questions about appropriate disclosure and patients’ “right not to know.”
Implementation	Intellectual property and commercialization	Patentability and commodification	The transformation of donor materials into patented biotechnological artifacts raises questions about the fair distribution of benefits. Restrictive intellectual property practices can hinder innovation and limit accessibility to technology and state-of-the-art research.
	Distributive justice	Unfair outcomes	High costs of precision therapies can limit accessibility, exacerbating health inequities among socio-economic groups and healthcare systems. Stratification can potentially result in genetic discrimination.

## Phase I: Research

Tissue chip research is primarily situated in the preclinical phase when these models are created, tested, and optimized to be scientifically robust and potentially translatable to clinical or commercial applications. The reviewed literature mentioned five issues relevant to tissue chip research: privacy and confidentiality, informed consent, evidence, animal experimentation, and ontology and moral status.

### Privacy and confidentiality

Tissue chip research relies on biomaterials and aggregated datasets from biobanks. It also integrates diverse data types (e.g., phenotypic, genetic, and demographic) to increase its therapeutic utility in PM applications (Ingber, 2022). These practices raise ethical questions that intersect with broader challenges in biomedical and bioethical research. They reflect the principle of respect for autonomy, which emphasizes the importance of individual control over how biological materials are used, especially in secondary or shared research contexts; non-maleficence, which addresses the risk of harm from data misuse, including breaches of confidentiality or re-identification; and justice, which is implicated when sensitive information is disclosed to third parties in a way that may lead to material or immaterial harm. This section examines how insights from organoids, PM, and DTs can help contextualize these broader ethical challenges for tissue chip research.

In our literature sample, discussions predominantly focus on genetic or informational privacy, that is, the right to protection from unauthorized disclosure of personal health information (Brothers and Rothstein, 2015; Lunshof et al., 2008). Informational privacy is closely linked to confidentiality, which indicates the practices that restrict access to this information to specifically authorized recipients (Adjekum et al., 2017; Kinkorová, 2016; Ormond and Cho, 2014; Williams and Anderson, 2018; Winkler and Knoppers, 2022). It is argued, however, that these principles face challenges in the context of genomics and biobank research, where the increased reliance on collecting, sharing, and integrating data from large and diverse datasets—including biospecimens, omics, clinical documentation in electronic health records (EHRs), and lifestyle information—raises concerns about the potential for unauthorized access, misuse, and the re-identification of anonymized data (Brothers and Rothstein, 2015; Juengst et al., 2016; Obafemi-Ajayi et al., 2022; Rothstein, 2021; Sharon, 2017).

It is argued, for example, that genetic or biobank data are interesting for third parties such as insurance companies, employers, and law courts due to their potential to reveal insights about an individual’s health and risk status (Bassil and Horstkötter, 2023; Boers and Bredenoord, 2018; Boers et al., 2016, 2018, 2019; Bollinger et al., 2021; Bredenoord et al., 2017; Bukreeva et al., 2024; Capasso et al., 2024; Farahany et al., 2018; Harris et al., 2022; Hartung et al., 2023; Hyun et al., 2020; Kataoka et al., 2024a, 2024b; Lavazza, 2019; Lavazza and Chinaia, 2023; Lensink et al., 2020, 2021a, 2021b; Lewis and Holm, 2022; MacDuffie et al., 2023; Mollaki, 2021; Munsie et al., 2017; Ravn et al., 2023; Stoeklé et al., 2021). Specifically, it is stated that the inappropriate disclosure of this information to such parties could result in material harm (e.g., loss of insurance) and immaterial harm (e.g., stigma or discrimination) (Ahmed et al., 2023; Browman et al., 2014; Carnevale et al., 2023; Chow-White et al., 2015; DuBois et al., 2021; Egalite et al., 2014; Evans, 2017; Farasati Far, 2023; Feiler et al., 2017; Green et al., 2023; Hazin et al., 2013; Lysaght et al., 2020; Müller et al., 2020; Popa et al., 2021; Rainey, 2022; Rauter et al., 2021; Rothstein, 2021; Schaefer et al., 2020; Sharon, 2017; Sui et al., 2023; Toh et al., 2021; Vaszar et al., 2003; Vos et al., 2017). Additionally, several authors argue that the potential misuse of personal information can erode trust relations between the public and healthcare systems (Adjekum et al., 2017; Ahmed et al., 2023; Dion-Labrie et al., 2010; Effy et al., 2018; Geneviève et al., 2023; Goncharov et al., 2022; Green et al., 2023; Hollister and Bonham, 2018; Lee, 2021a, 2021b; Lee et al., 2019a, 2019b; Lysaght et al., 2020; Meslin and Cho, 2010; Minari et al., 2018; Myskja and Steinsbekk, 2020; Obafemi-Ajayi et al., 2022; Ong et al., 2021; Rauter et al., 2021; Thapa and Camtepe, 2021; Williams and Anderson, 2018). Lack of trust and fear of inappropriate disclosure may, in turn, cause patients to withhold critical information and forego timely treatment, ultimately compromising healthcare quality and scientific development (Brothers and Rothstein, 2015; Farasati Far, 2023).

Although considerable effort is devoted to improving data safety, absolute privacy and confidentiality may be impossible to guarantee in modern biomedical research (Adjekum et al., 2017; Ahmed et al., 2023; Browman et al., 2014; Bruynseels et al., 2018; Chow-White et al., 2015; Delpierre and Kelly-Irving, 2018; Effy et al., 2018; Erdmann et al., 2021; Evans, 2017; Farasati Far, 2023; Fusar-Poli et al., 2022; Goncharov et al., 2022; Hazin et al., 2013; Juengst and Van Rie, 2020; Kinkorová, 2016; Lee et al., 2019a; Lewis et al., 2014; Lunshof, 2006; Lysaght et al., 2020; McGowan et al., 2014; Ormond and Cho, 2014; Rothstein, 2021; Safarlou et al., 2023; Schaefer et al., 2019; Schleidgen and Marckmann, 2013; Shoaib et al., 2017; Shoenbill et al., 2014; Stratton and Olson, 2023; Sui et al., 2023; Thapa and Camtepe, 2021; Vaszar et al., 2003; Williams and Anderson, 2018; Winkler and Knoppers, 2022). For instance, a common strategy to protect privacy is data pseudonymization. However, individuals can be re-identified by cross-referencing genetic information from samples with other datasets using advanced big data analytics (Boers and Bredenoord, 2018; Boers et al., 2016; Browman et al., 2014; Chow-White et al., 2015; de Jongh et al., 2022; Winkler and Knoppers, 2022). Furthermore, it is argued that fully anonymized datasets could hinder the validation of predictive models, as researchers cannot verify whether these models accurately reflect real patient outcomes (Boers and Bredenoord, 2018; Boers et al., 2016, 2019; Bredenoord et al., 2017; de Jongh et al., 2022; Lensink et al., 2020, 2021a). Absolute anonymization might also prevent research benefits from being returned to donors (Boers and Bredenoord, 2018; Boers et al., 2016; Bredenoord et al., 2017).

These considerations indicate the need for clear data governance in tissue chip research that protects patient privacy while allowing for the secure sharing of de-identified datasets needed to validate and innovate tissue chip models (U.S. Government Accountability Office, 2025). When research involves the transfer of biomaterials and data to/from non-European countries, researchers must comply with the provisions defined in Directive 2004/23/EC and consider the General Data Protection Regulation (GDPR) (CEN/CENELEC, 2024).

### Informed consent

Informed consent is required for any type of research that involves human participation and the use of biospecimens or personal information. Informed consent involves three core elements: adequate information, voluntariness, and competence (Kinkorová, 2016; Lee, 2021a). This means that, before consenting, subjects participating in (medical) research must be adequately informed about and understand the purpose and potential risks of the research project, as well as the possibility to refuse or withdraw participation at any time without consequences (Kinkorová, 2016; Mollaki, 2021). Informed consent considerations in tissue chip research primarily reflect the principle of autonomy, which requires that participants are given meaningful choice over how their biological material and personal data are used; non-maleficence, which raises questions about protecting participants from unforeseen and potentially harmful uses of their data; and justice, which concerns the institutional obligations to safeguard participant interests over time, particularly in broad consent frameworks. This section examines how organoid, PM, and DT literatures contextualize consent-related challenges in tissue chip research.

PM and DT programs collect relevant information from large and diverse sources, including omics data, clinical documentation, EHRs, and lifestyle information, to create personalized treatments and prevention strategies. It is argued, however, that the sheer volume and complexity of these data can limit the ability of participants to fully understand how their material will be used (Goncharov et al., 2022; Hansson, 2010; Kuchinke, 2013; Lee, 2021a; Lunshof, 2006; Lunshof et al., 2008; McGowan et al., 2014; Parra-Calderón et al., 2018; Rainey, 2022; Spector-Bagdady et al., 2022; Stratton and Olson, 2023; Thakar and Fenton, 2023; Thapa and Camtepe, 2021; Tigard, 2021; Vaszar et al., 2003; Vos et al., 2017; Winkler and Knoppers, 2022). Moreover, the specific uses of data and biomaterials are not always predictable at the time of collection, which raises further questions as to whether consent can be truly informed (Boers and Bredenoord, 2018; Boers et al., 2018; Bredenoord et al., 2017; de Jongh et al., 2022; Kataoka et al., 2024a; Lensink et al., 2021b; Lewis and Holm, 2022; Mollaki, 2021).

In response to traditional consent, which assumes that participants can fully understand how their materials will be used, several alternative consent models have been proposed to better accommodate the diverse contexts and requirements of advanced biomedical research. These include specific, tiered, dynamic, broad, blanket, opt-in, opt-out, open, and governance consent, each with its strengths and weaknesses (see Table 2; Boers and Bredenoord, 2018; de Jongh et al., 2022; Lee, 2021a). The suitability of these consent models also depends on the stage or context of research. When biomaterials remain closely tied to donors, as in (personalized) clinical contexts, or when patients want to retain control over their material, some argue that high participant engagement in the form of specific, tiered, or dynamic consent may be most appropriate (Goncharov et al., 2022; Isasi et al., 2024; Lee, 2021a; Stratton and Olson, 2023; Thapa and Camtepe, 2021; Vos et al., 2017; Winkler and Knoppers, 2022; Wouters et al., 2021). Conversely, in broader applications such as high-throughput drug screening or population-scale research, the connection between donors and their material becomes less pronounced. In these cases, the material is effectively treated as anonymized and often used across multiple studies, and the need for re-consent for every new study is argued to become impractical (Vos et al., 2017; Winkler and Knoppers, 2022). In such contexts, broad or blanket consent may become more desirable. Importantly, it is stated that in broad consent procedures, the responsibility shifts to institutions, ethics committees, and researchers, who must now act as fiduciaries for patients’ interests (Goncharov et al., 2022; Lee, 2021a; Lensink et al., 2020; Vos et al., 2017; Winkler and Knoppers, 2022).

**Table 2:** Contemporary consent models with their strengths and weaknesses

Approach		Strengths	Weaknesses
Specific and re-consent	Consent is granted for a single and clearly defined study and scope. For additional uses, donors must be recontacted to provide new consent.	Respects individual values and donor autonomy.	Reconsenting can be resource intensive. It can also limit research efficiency.
Broad	Consent is granted for a broad range of future research uses, often unspecified at the time of consent. This provides researchers with more flexibility to use biospecimens without having to recontact donors.	Research flexibility and efficiency.	Limited engagement with donors; donors have little control of the future use of their tissue.
Dynamic	Utilizes digital communication platforms to enable ongoing, two-way interaction between participants and researchers. Participants can make decisions about specific uses of their biospecimens and data in real time.	Allows ongoing and flexible engagement with donor preferences.	Reconsenting can be resource intensive; requires complex infrastructures for effective communication.
Tiered	Participants are provided with a set of options, allowing them to specify the types of research for which their biospecimens and data can be used.	Respects individual values and donor autonomy.	Complex consent process; could limit the scope of research if donors restrict certain types of use.
Blanket	Donors provide unrestricted consent for their biospecimens and data to be used in all future research without limitations on research type or scope.	Research flexibility and efficiency.	Limited engagement with donors; donors have minimal control of the future use of their tissue.
Opt-in	Explicit consent is required for each individual to allow their samples to be used in research.	Respects individual values and donor autonomy.	Resource intensive: requires (re)contacting donors for new scientific use.
Opt-out	Consent is presumed unless participants explicitly decline.	Increases flexibility and efficiency.	Assuming consent can compromise donor autonomy and engagement.
Open	Donors consent to the unrestricted re-disclosure of personal information, including incidental findings. Anonymity, privacy, or confidentiality are not promised	Transparent and honest. Promotes the collaborative nature of contemporary science.	Acknowledging privacy risks might deter potential study participants.
Governance	Donors consent to governance structures that protect their long-term interests. Participants are continuously informed about revisions made in these governance structures.	Allows ongoing engagement with donor preferences.	Reconsenting can be resource intensive; requires complex infrastructures for effective communication.

These challenges suggest that tissue chip research should adopt consent models that balance meaningful donor choice with the increasing needs of large-scale, translational research. Where relevant, donors should be informed that their samples and data may be used not only for academic studies but also to validate tissue chip models for potential clinical and regulatory use. Using dynamic or tiered consent models, paired with clear communication about these possible future uses, can help reconcile donor autonomy with advancing tissue chip systems toward clinical and regulatory implementation. These and similar expectations are consistent with the ethical and legal frameworks outlined in the Declaration of Helsinki, the GDPR, and Directive 2004/23/EC (CEN/CENELEC, 2024).

### Evidence

Tissue chips are predictive models that can be used to inform and justify (clinical) decisions. As such, they raise broader ethical and epistemological questions about the nature, quality, and reliability of the evidence they produce. The literature in our sample discusses three types of potentially unreliable evidence: inconclusive, misguided, and inscrutable (Mittelstadt et al., 2016), as well as their associated ethical challenges. These concerns reflect non-maleficence, when flawed or unreliable predictions risk causing harmful clinical decisions or when models fail to identify outcomes that could support safe and effective care; justice, when models underperform for certain demographic groups; and autonomy, when patients and clinicians can neither understand nor explain the inner workings of a model, effectively undermining meaningful choice and shared decision-making. This section considers these challenges to further contextualize the ethical use of tissue chips in biomedical research and clinical decision-making.

Firstly, models that offer probable yet uncertain knowledge may generate inconclusive evidence. In drug testing or clinical medicine, it is argued that these results might lead to ineffective or harmful recommendations (Ahmed et al., 2023; Braun, 2021; Browman et al., 2014; Delpierre and Kelly-Irving, 2018; Effy et al., 2018; Emmert-Streib and Yli-Harja, 2022; Fusar-Poli et al., 2022; Gefenas et al., 2011; Giusti, 2021; Green et al., 2023; Hey and Barsanti-Innes, 2016; Korngiebel et al., 2017; Lee, 2009, 2021b; Lewis et al., 2014; McClellan et al., 2013; Popa et al., 2021; Shoenbill et al., 2014; Vogt and Hofmann, 2022). While tissue chips aim to recapitulate human physiology for evaluating compound toxicity and benefit-risk profiles, they fail to capture the full complexity of the human body (Leung et al., 2022). This limitation raises important questions about the standards of model reliability that must be met in tissue chip research—as well as other models, including animals—to ensure their predictions are sufficiently accurate to make safe clinical decisions. What level of accuracy is ethically acceptable when using tissue chip data to inform or justify potentially high-risk clinical interventions (Mittelstadt, 2021)?

Secondly, misguided evidence can occur when bias in the input data leads to skewed results or predictions that might disproportionately affect underrepresented populations (Boers et al., 2018; Braun, 2022; Brothers and Rothstein, 2015; Browman et al., 2014; Callier, 2019; Carnevale et al., 2023; Clark et al., 2024; Cohn et al., 2017; Dankwa-Mullan et al., 2025; Erikainen and Chan, 2019; Ferlito et al., 2024; Fleck, 2022; Fusar-Poli et al., 2022; Geneviève et al., 2023; Goncharov et al., 2022; Green et al., 2023; Greenfield, 2024; Hazin et al., 2013; Hendricks-Sturrup et al., 2024; Hollister and Bonham, 2018; Iqbal et al., 2022; Knoppers and Avard, 2009; Lee, 2009, 2021b; Lee et al., 2019a, 2019b, 2025; Lensink et al., 2021b; Lunshof, 2006; Mao et al., 2024; McClellan et al., 2013; Mensah et al., 2019; Obafemi-Ajayi et al., 2022; Ormond and Cho, 2014; Rezzani et al., 2019; Sabatello, 2018; Schaefer et al., 2019, 2020; Shemie et al., 2021; Sierra-Mercado and Lázaro-Muñoz, 2018; Skantharajah et al., 2023; Tabor and Goldenberg, 2018; Tranvåg et al., 2021; Viaña, 2024; Vos et al., 2017; Williams and Anderson, 2018; Wouters et al., 2021). Tissue chips rely on biomaterials, and a lack of diversity in these materials could limit the understanding we have of particular pathologies or lead to the creation of products that are only accessible or effective to certain demographics. There are well-documented biological differences between ethnic groups and genders that might affect how pharmaceutical compounds and medical devices perform, including factors like bone mineral density (Wagner and Heyward, 2000), skin integrity (Wesley and Maibach, 2003), heart rate variability and blood pressure regulation (Hill et al., 2021), lung capacity (Bellemare et al., 2003), and hormone secretion (Farkouh et al., 2020; Sheth et al., 2015). If these variables are inadequately represented in biobank studies and tissue chip research, the models could fail to predict accurate responses across populations.

A notable discrepancy can be observed in how diversity and inclusivity are addressed across organoid, PM, and DT research. While PM and DT research emphasizes the importance of integrating diversity into datasets to improve model quality and accessibility, the organoid ethics literature largely neglects these considerations, with one paper mentioning them only in passing (Lewis and Holm, 2022).

Thirdly, model complexity can make it difficult to understand or explain how specific predictions are generated, leading to inscrutable evidence. This can undermine confidence in the produced evidence and pose challenges for reproducing, standardizing, and extrapolating results (Carnevale et al., 2023; Chow-White et al., 2015; Delpierre and Kelly-Irving, 2018; Knox and Svendsen, 2023; Rauter et al., 2021; Rothstein, 2021; Schaefer et al., 2019; Truby and Brown, 2021). This challenge might be especially pronounced in body or multi-organ chip systems that seek to model whole-body-level physiologies to predict pharmacokinetic and pharmacodynamic parameters (Ingber, 2022; Leung et al., 2022). However, if the mechanisms underlying these predictions (e.g., cellular interactions or biochemical pathways affecting progression and drug metabolism) remain opaque, it might become challenging to use tissue chip data as evidence for human biomedical decisions.

Some authors have suggested integrating tissue chips with computational models (Deng et al., 2023; Przekwas and Somayaji, 2020) and additional data streams such as eHealth metrics (van den Berg et al., 2019) to enhance their predictive capabilities. However, this integration may increase the potential for inscrutability as the interactions between these tools, data sources, and the tissue chip’s biological systems can be unpredictable and difficult to reproduce.

These challenges suggest that tissue chip research and implementation should prioritize clear evidence standards and equitable model validation to support safe clinical use (U.S. Government Accountability Office, 2025). Regulators and researchers should collaboratively define minimum evidence thresholds, terminology, and reporting standards for tissue chip models used in preclinical testing and clinical decision-making (CEN/CENELEC, 2024). Additionally, systematic efforts to improve diversity in biobank samples used for tissue chip development can help address bias and support equitable model performance across populations.

### Animal experimentation

Animal models have been instrumental in making medical and scientific advancements, but their use has long raised ethical concerns, particularly regarding animal welfare and ethical frameworks like the 3Rs of reducing, refining, and replacing the use of animals. While testing new pharmaceutical compounds remains a necessary step in translational and clinical research (Ingber, 2020, 2022), tissue chips and organoids have emerged in the literature as a long-awaited alternative to animal models with the potential to revolutionize traditional evidence-based research methodologies (Bredenoord et al., 2017; Hu and Yang, 2023; Ingber, 2020, 2022; Lee et al., 2022; Wilkinson, 2019). This section examines how tissue chips and related technologies may impact the use of animals in (bio)medical research and considers the ethical and regulatory debates surrounding their potential to replace or complement animal models.

Firstly, it is argued that the emergence of advanced *in vitro* models like tissue chips and organoids can considerably affect the ethical debate on animal experimentation (Bredenoord et al., 2017; Ding et al., 2022; Doke and Dhawale, 2015; Hartung et al., 2023; Hyun et al., 2020; Jowitt, 2023; Kataoka et al., 2023; Koplin, 2024; Lee et al., 2022; Mollaki, 2021; Presley et al., 2022; Smirnova et al., 2023; Stoeklé et al., 2021; Wilkinson, 2019). Compared to animal models, tissue chip systems and organoids offer the translational advantage of being developed directly from human cells, thereby avoiding many translational gaps caused by species differences (Ingber, 2020, 2022). Nonetheless, recent trends indicate a substantial rise in animal use, with numbers increasing significantly over the past decade (Hu and Yang, 2023; Park et al., 2024). The continued reliance on animal models is deemed unethical by some due to concerns about animal welfare and the potential for suffering and death (Bredenoord et al., 2017; Hu and Yang, 2023; Hyun et al., 2020; Lee et al., 2022; Park et al., 2024; Stoeklé et al., 2021). In response, it is argued that the use of animals can be accepted but only under strict conditions. A set of principles summarized as the 3Rs is often used as a policy tool to strike a balance between allowing animal experimentation and respecting their lives and well-being (Bredenoord et al., 2017; Erler, 2024; Hu and Yang, 2023; Lee et al., 2022; Park et al., 2024; Sharoni, 2024; Wilkinson, 2019).

Whether these models will completely replace the use of animals remains an open question (Bredenoord et al., 2017; Ingber, 2020). Nonetheless, it is argued that they could complement, rather than conflict with, traditional research methodologies. Some authors, for instance, advocate for a “comply or explain” paradigm, where researchers must either utilize alternative *in vitro* or *in silico* methods or provide a justification for the use of animals (Bredenoord et al., 2017).

Secondly, it is argued that more conclusive guidelines and criteria are required to assess the potency of tissue chips and organoids to replace animals as tools for drug testing and development. In this context, initial responses tend to focus on defining what constitutes viable evidence for demonstrating the ability of tissue chips to recapitulate human physiological responses and pathophysiology accurately (Hu and Yang, 2023; Ingber, 2022; Lee et al., 2022; Leung et al., 2022; Low et al., 2021; Park et al., 2024; Wilkinson, 2019). Others argue that integrating tissue chips and related advanced *in vitro* models into mainstream scientific research also depends on considering concepts such as awareness, access, and education (Sharoni, 2024). Here, it is believed that reflecting on these notions promotes ethical responsibility in biomedical innovation.

Lastly, for tissue chips to serve as a true replacement or companion to animal models, the field must overcome key challenges, including the need for robust standardization, reproducibility, and quality control (Leung et al., 2022; Low et al., 2021). It is argued that addressing these challenges requires multi-institutional and interdisciplinary collaboration (Bredenoord et al., 2017; Hyun, 2017; Hyun et al., 2020; Sample et al., 2019; Smirnova et al., 2023), as well as regulatory authority evaluation. Additionally, some argue that maintaining ethical and social responsibility requires adopting an “embedded ethics” approach (Hartung et al., 2023; Lavazza and Chinaia, 2023; Lensink et al., 2020). This would help ensure that the development and implementation of tissue chips occur in an ethically and socially responsible way.

To reduce animal use and adopt validated new approach methods (NAMs) in regulation, researchers and regulators need clear strategies for building and evaluating these approaches. The US Food and Drug Administration (FDA) has started facilitating this by issuing recommendations to create a central NAM office, develop impact metrics, set a uniform qualification framework, ensure rigorous review of NAM-based applications, invest in high-impact NAM initiatives, and maintain a central agency-wide database (Han, 2023; U.S. Food and Drug Administration, 2024).

### Ontology and moral status

Considerations concerning ontology and moral status, respectively, relate to what an entity is—i.e., how it fits into existing categories of living and non-living entities—and how this informs the extent to which the entity deserves ethical respect or special treatment. In this context, key considerations include the potential of advanced *in vitro* models to develop attributes such as consciousness and sentience (Bayne et al., 2020, 2024; Boyd and Lipshitz, 2024; Chen et al., 2019; de Kerckhove, 2021; Harris et al., 2022; Hostiuc et al., 2019; Hyun et al., 2020; Jeziorski et al., 2023; Jowitt, 2023; Kataoka et al., 2023, 2024a, 2024c, 2024d; Koplin and Gyngell, 2020; Koplin and Savulescu, 2019; Kreitmair, 2023; Lavazza, 2019, 2020, 2021a, 2021b; Lavazza and Chinaia, 2023, 2024; Lavazza and Massimini, 2018; Lavazza and Pizzetti, 2020; Lavazza and Reichlin, 2023; Lewis and Holm, 2022; McKeown, 2023; Milford et al., 2023; Montoya and Montoya, 2023; Niikawa et al., 2022; Ota et al., 2024; Pichl et al., 2023; Presley et al., 2022; Sawai et al., 2019, 2022; Sawai and Kataoka, 2024; Shepherd, 2018), their ability to emulate characteristics of early-stage embryonic structures (Bredenoord et al., 2017; Hostiuc et al., 2019; Lavazza, 2021b; Lensink et al., 2020; Lewis and Holm, 2022; Mollaki, 2021; Munsie and Gyngell, 2018; Munsie et al., 2017; Sawai and Kataoka, 2024; Simons et al., 2024), and the potential for cross-species reproduction and the “humanization” of animals in chimera research (Barnhart and Dierickx, 2022; Bassil and Horstkötter, 2023; Blumenthal-Barby, 2024; Boyd, 2024; Chen et al., 2019; Ding et al., 2022; Farahany et al., 2018; Hartung et al., 2023; Hinterberger and Bea, 2023; Hyun et al., 2020; Jowitt, 2023; Kataoka et al., 2023; Koplin and Massie, 2021; Koplin and Gyngell, 2020; Lavazza, 2020, 2021b; Lavazza and Chinaia, 2023; Lavazza and Reichlin, 2023; Lewis and Holm, 2022; Mollaki, 2021; Munsie et al., 2017; Presley et al., 2022; Sample et al., 2019; Sawai et al., 2019, 2022; Stoeklé et al., 2021). Other themes discussed in the literature include the hybrid status of advanced *in vitro* models, as well as their potential to provoke hype and false hope. These issues interact with non-maleficence when the capacity for suffering demands moral restraint, with beneficence when such models offer pathways for studying severe or otherwise inaccessible conditions, and with autonomy when donors’ emotional attachment or identification with the models complicates consent and ongoing participation. This section further explores these issues and how they relate to tissue chip research.

Questions about ontology and moral status apply most directly to tissue chips designed for recapitulating the neural activity and structure of specific brain regions to study psychiatric or neurodegenerative conditions like Alzheimer and autism (Servais et al., 2024). Most importantly, if these models were to acquire the ability to experience, e.g., pain, pleasure, or distress, they might require the rights and considerations currently afforded to sentient animals. Researchers generally doubt that brain models will ever attain the complexity needed for consciousness or sentience (Bayne et al., 2020; Hostiuc et al., 2019; Koplin, 2024; Lavazza, 2021b; Lavazza and Chinaia, 2023; Lavazza and Pizzetti, 2020; Sawai et al., 2022). It is also argued that overly strict regulations in this area could hinder research and delay breakthroughs in understanding and treating severe mental health disorders and neurodegenerative diseases (Hyun et al., 2020; Koplin and Savulescu, 2019; Lavazza, 2020; McKeown, 2023; Presley et al., 2022). In response, different approaches have been developed for monitoring this type of research, with multiple suggestions for implementing specialized review boards, frameworks, and legislation to prevent unethical use, while still facilitating scientific innovation (Bayne et al., 2020; Hostiuc et al., 2019; Hyun, 2017; Koplin and Massie, 2021; Koplin and Gyngell, 2020; Koplin and Savulescu, 2019; Lavazza, 2021b; Lavazza and Massimini, 2018; Lavazza and Pizzetti, 2020; Sawai et al., 2019, 2022).

Secondly, it is argued that advanced models like organoids or tissue chips possess both “object”- and “subject”-like values. On the one hand, they are tools designed for research and innovation; on the other, they closely resemble human physiology. As such, tissue chips and similar models can be seen as *hybrids*—entities whose ontological and moral status remains ambiguous (Boers et al., 2019; Lavazza, 2019; Sample et al., 2019; Vogt and Hofmann, 2022). This hybrid status might provoke a strong sense of personal attachment or identification and influence how donors feel about the use of their material in research (Boers et al., 2018, 2019; Dam and Green, 2022; Lensink et al., 2021b; Lewis and Holm, 2022; Lupton, 2021; Nyholm, 2021; Yokoro, 2023).

Lastly, advanced predictive models may provoke hype and false hope. This issue is primarily connected to how the media communicates about the moral status and potential for consciousness in these models. Recent reports indicate that media interest in brain organoid research has grown significantly, with only 15 published reports in 2013–2016 and 45 in 2019 alone (Bassil and Horstkötter, 2023; Presley et al., 2022). However, multiple authors express concern that excessive media attention could lead to exaggerated and unrealistic expectations about what these models can achieve (Bassil and Horstkötter, 2023; Boers and Bredenoord, 2018; Bollinger et al., 2021; Bredenoord et al., 2017; Chen et al., 2019; Hartung et al., 2023; Hyun et al., 2020; Ide et al., 2021; Iltis et al., 2023; Lavazza and Chinaia, 2023, 2024; Lensink et al., 2021a; Munsie et al., 2017; Presley et al., 2022; Ravn et al., 2023; van Till and Bunnik, 2024). This might foster false hopes or unnecessary worry, contribute to mistrust in science, skew funding priorities, and divert resources away from other important areas of research (Chen et al., 2019; Flaum et al., 2018; Hyun et al., 2020; Ide et al., 2021; Lavazza and Chinaia, 2024; Lensink et al., 2021a; Munsie et al., 2017; Presley et al., 2022; Thakar and Fenton, 2023; van Till and Bunnik, 2024).

## Phase II: Clinical testing

Integrating tissue chips into clinical settings could (re)shape certain aspects of general medical care by introducing new tools for precision diagnostics and treatments. However, this integration also brings about several challenges related to evidence standards and effects on patient care.

### Evidence standards in translational research

The primary commercial and/or clinical applications of tissue chips focus on profiling the toxicity of compounds in human tissues and on *in vitro* disease modeling to investigate disease mechanisms, therapeutic efficacy, and potential off-target effects (Low et al., 2021). However, by offering more human-relevant and personalized data, tissue chips introduce new evidence types that challenge traditional trial designs and regulatory standards. In this context, the sampled literature discusses various topics relevant to tissue chips, including the implications of first-in-human (FIH) trials, the dual-purpose potential of *in vitro* models in research and care, the use of *n = 1* evidence (i.e., individual-specific data rather than findings based on large cohorts), and the risks of overdiagnosis. These raise ethical questions related to non-maleficence, which applies when inaccurate predictions or premature clinical use of tissue chip-based products could cause harm; beneficence, when the potential for more precise or effective treatments supports their use in contexts where traditional evidence standards are hard to meet; and justice, when unequal access or the exclusion of rare disease populations leads to unfair outcomes. This section contextualizes these issues to inform the transition of tissue chips from research to clinical applications.

Firstly, tissue chips are expected to play a significant role in high-throughput screening for predicting the absorption, distribution, metabolism, and excretion of novel and existing pharmaceutical compounds (Ingber, 2022; Low et al., 2021; van den Berg et al., 2019). By providing human-relevant testing conditions, tissue chips might complement or replace other preclinical models—like animals—and thus reduce the potential for risk to human subjects in FIH trials. However, tissue chips cannot entirely replace human testing, as novel drugs must ultimately be tested on human subjects to evaluate safety and efficacy. Multiple authors argue that FIH trials require robust preclinical evidence to demonstrate safety, efficacy, and reliability, as well as a favorable risk-benefit ratio that justifies the intervention (Bredenoord et al., 2017; Harris et al., 2022; Hyun et al., 2020; Mollaki, 2021; Schneemann et al., 2020; Sharrer, 2012). Furthermore, ethical concerns arise regarding the inclusion of vulnerable populations, like children, in FIH trials (Avard et al., 2009; Braun and Krutzinna, 2022; Mollaki, 2021; Schneemann et al., 2020). It is argued that in such cases the principle of subsidiarity should be considered, dictating that research involving children is permissible only when equivalent studies cannot be conducted on adults (Schneemann et al., 2020).

Secondly, it is argued that advanced biotechnologies can blur the line between research and clinical care (Boers et al., 2016, 2019; Bredenoord et al., 2017; Dam and Green, 2022; Lensink et al., 2020, 2021a). Tissue chips exemplify this dual-purpose potential. For example, a liver-on-a-chip could model drug metabolism in research settings, while simultaneously informing personalized treatment strategies by predicting individual responses to specific therapies. This overlap raises questions about which regulatory and reimbursement standards apply (Boers et al., 2016; Bredenoord et al., 2017). Should personalized tissue chips be regulated by more lenient research-based policies and protocols, or should they follow the stricter standards required in clinical care?

Thirdly, tissue chips may challenge existing regulatory frameworks by providing *n = 1* evidence (Boers et al., 2016; Bredenoord et al., 2017; de Jongh et al., 2022; Vogt and Hofmann, 2022). Specifically, by recapitulating disease models with patient-derived primary or iPSC cells, it becomes possible to stratify individual patients into distinct subpopulations most likely to benefit from certain therapeutic regimens. A precursor to this development is the use of gut organoid swelling assays to screen for treatment efficacy in rare diseases like cystic fibrosis (CF), which are now routinely employed to help identify patients who would benefit from—and could be reimbursed for—expensive CF treatments (Mummery et al., 2021). Such approaches might also lead to the development of individual “you-on-a-chip” models for patients with rare cancers or (genetic) diseases (Ingber, 2022; Leung et al., 2022; Low et al., 2021). In recent CF research, for example, patient-derived cells were utilized to develop a pancreas-on-chip model to understand the CF transmembrane conductance regulator protein and its role in insulin secretion (Low et al., 2021; Shik Mun et al., 2019). *N = 1* evidence procedures could substantially benefit patient populations affected by such rare diseases, specifically as these populations are often too small to make large clinical trials economically or statistically feasible. However, it is argued that regulatory agencies may find it difficult to evaluate such tissue chip data because *n = 1* studies lack the predictive validity and generalizability traditionally required for approval (Boers et al., 2016).

Lastly, predictive models could provide early warnings by enabling detailed analyses of disease processes and biomarker detection. While early risk detection can be beneficial, it is argued that it might also lead to overdiagnosis (Bruynseels et al., 2018; Carter et al., 2016; Erikainen and Chan, 2019; Feiler, 2019; Green et al., 2023; Huang et al., 2022; Hummel and Braun, 2020; Juengst and McGowan, 2018; Kerr et al., 2018; McGonigle, 2016; Mittelstadt, 2021; Morley and Floridi, 2020; Pellé and Nurock, 2012; Popa et al., 2021; Savard, 2013; Sharon, 2017). Most fundamentally, overdiagnosis occurs when individuals are diagnosed with and treated for conditions that would not have caused significant symptoms or harm throughout their lifetime. In this context, several authors claim that the growing ability to access and analyze vast amounts of personal health information can redefine what is considered “normal” or “healthy” (Bruynseels et al., 2018; Cho and Martinez-Martin, 2023; Huang et al., 2022; Iqbal et al., 2022; Pellé and Nurock, 2012; Savard, 2013). This carries the risk of classifying natural or harmless variations as conditions in need of medical attention. Additionally, some caution that overemphasizing informed choice in managing decisions about such variations may lead to excessive health surveillance (Braun and Krutzinna, 2022; Erikainen and Chan, 2019; Feiler, 2019; Huang et al., 2022; Hummel and Braun, 2020; Juengst and McGowan, 2018; Morley and Floridi, 2020; Popa et al., 2021; Savard, 2013; Sharon, 2017; Truby and Brown, 2021), which could shift responsibility from physicians to patients and potentially cause anxiety, regret, or guilt (Carter et al., 2016; Chadwick and O'Connor, 2013; Huang et al., 2022; Kerr et al., 2018).

To facilitate the responsible use of tissue chip data in clinical contexts, regulatory bodies, researchers, and clinicians can draw on existing FDA frameworks such as the tiered criteria under 21 CFR 1271.10(a) for human cells, tissues, and cellular- and tissue-based products to define when tissue chip data are sufficiently robust to inform clinical decisions, guide FIH trials, and support *n = 1* personalized applications without compromising patient safety. Additionally, further criteria should specify how to distinguish predictive signals that justify intervention from those likely to lead to overdiagnosis.

### Patient care

Integrating tissue chips into clinical practice introduces various practical and ethical questions about their impact on healthcare delivery. The sampled literature discusses three key issues capable of affecting patient care: the clinical expertise required to use tissue chips effectively, which relates to beneficence in ensuring competent care; the demands on training, staffing, and counseling, which implicate justice when access to these recourses is unequal; autonomy in supporting patients’ understanding and respecting their right to decline unwanted information; and the emergence of incidental findings, which engages non-maleficence when its disclosure may cause psychological harm.

First, as tissue chips enter clinical use, an important question is whether physicians possess the necessary expertise required to deliver tissue chip-based treatments. Some authors claim that formulating prevention and treatment strategies based on patient-specific bioassays and organ-level interactions might require specialized knowledge that some physicians currently lack, which raises questions about the potential for errors, legal liabilities, and the (in)ability of physicians to meet the more demanding standards of personalized care (Brothers and Rothstein, 2015; Effy et al., 2018; Juengst and McGowan, 2018; Myskja and Steinsbekk, 2020; Sharrer, 2017; Vogt and Hofmann, 2022; Vos et al., 2017). Furthermore, some authors argue that the effective integration of tissue chips into clinical practice may demand substantial staffing, resources, and extra training, which some healthcare systems cannot afford (Brothers and Rothstein, 2015; Shoenbill et al., 2014; Vos et al., 2017; Zarif, 2022).

Secondly, it is argued that the complexity of advanced medical technologies is likely to impact patient care by increasing demands on physicians’ time and expertise (Gefenas et al., 2011; Wouters et al., 2021). A responsible approach to ensure that individuals make deliberate and well-informed decisions requires that they fully understand the implications of a treatment. Dedicated counseling plays a critical role in this process. However, multiple authors claim that when large populations are routinely exposed to personalized testing or tissue chip-based diagnostics, the demands on healthcare facilities in terms of staffing and resources could make it challenging to uphold these standards (Ahmed et al., 2023; Blumling et al., 2021; Browman et al., 2014; Dion-Labrie et al., 2010; Erdmann et al., 2021; Gefenas et al., 2011; Guadalajara et al., 2022; Hazin et al., 2013; Juengst et al., 2016; Knoppers and Avard, 2009; Myskja and Steinsbekk, 2020; Regniault et al., 2009; Sabatello et al., 2018; Safarlou et al., 2023; Sharon, 2017; Shoaib et al., 2017; Stratton and Olson, 2023; Tuteja et al., 2013; Wouters et al., 2021).

Lastly, when tissue chips are designed to emulate and analyze an individual’s physiology, they may uncover previously unknown health risks, including genetic predispositions to cancer, cardiovascular conditions, or neurodegenerative diseases. It is argued that such incidental findings could reveal hereditary risks relevant to family members, which raises questions as to whether, how, and to whom this information should be disclosed, specifically as it can seriously impact an individual’s life and choices (Ahmed et al., 2023; Blasimme and Vayena, 2016; Bunnik et al., 2012; Erdmann et al., 2021; Finkler, 2017; Hazin et al., 2013; Hummel and Braun, 2020; Ormond and Cho, 2014; Shoenbill et al., 2014; Tan et al., 2024; Vaszar et al., 2003; Williams and Anderson, 2018; Winkler and Knoppers, 2022). According to some, the “right not to know” must be respected for individuals who prefer not to receive genetic or other test results (Erdmann et al., 2021; Ormond and Cho, 2014; Safarlou et al., 2023). At the same time, there is a growing consensus among experts that incidental findings should be disclosed only when they are clinically valid and actionable (Ormond and Cho, 2014; Shoenbill et al., 2014; Vos et al., 2017; Winkler and Knoppers, 2022).

To support responsible clinical use of tissue chips, regulators and healthcare institutions should develop guidance on physician training and qualifications, establish patient counseling protocols to ensure clear and actionable communication, and define when and how incidental findings should be disclosed. The FDA’s current good tissue practice requirements under 21 CFR 1271 Subpart D establish training and quality standards for tissue-based products. Recommendations from the Secretary’s Advisory Committee on Human Research Protections on returning research findings offer additional guidance for managing incidental findings.

## Phase III: Implementation

The implementation of tissue chips extends beyond research and clinical settings, specifically as it could affect society at large by (re)shaping general medical care, industry practices, and social perceptions. The implementation process of tissue chips raises ethical questions regarding intellectual property (IP) and commercialization and distributive justice.

### Intellectual property and commercialization

When biomaterials or data are transformed into biotechnological artifacts that are considered sufficiently novel, both the materials and technology used for their derivation and analysis become subject to patents and licensing agreements (Boers et al., 2016, 2019; de Jongh et al., 2022). This means that parties other than original donors (e.g., researchers or companies) can claim property rights over these artifacts or the methods used to produce them. While IP practices can drive scientific innovation and incentivize advancements in tissue chip technologies, they also raise critical questions about balancing the interests of donors, researchers, and commercial entities. This section examines the ethical considerations that surround the commodification of human tissue and the potential impact of IP restrictions on the development and accessibility of tissue chip-based innovations These developments raise questions about justice, where benefits are distributed inequitably; non-maleficence, where restrictive IP practices can reduce access, stifle collaboration, and degrade care quality; and autonomy, when commercial uses conflict with donors’ intentions or override the scope of their consent.

Firstly, the interdisciplinary character of tissue chip research creates a complex dynamics around balancing their different interests and rights. In this context, it is argued that the commodification of human tissue creates friction between the altruistic intentions of donors and the financial interests of commercial parties (Boers and Bredenoord, 2018; Boers et al., 2019; Bredenoord et al., 2017; de Jongh et al., 2022; Lensink et al., 2020, 2021a, 2021b; Lewis and Holm, 2022). Typically, individuals donate their material with the intended purpose of benefiting society, expecting no financial return. However, when companies use this material to create profitable products, such as novel drugs or diagnostic tools, it raises questions about the fair distribution of benefits (Boers et al., 2016, 2018, 2019; Hyun et al., 2020; Lavazza, 2019; Lensink et al., 2020, 2021a). According to some, this tension emphasizes the need for equitable distribution arrangements, such as reinvesting profits into further research or providing donors with non-monetary benefits like early access to novel therapies (Boers and Bredenoord, 2018; Boers et al., 2016, 2019; Hyun et al., 2020; Lensink et al., 2020, 2021b).

Secondly, multiple authors argue that IP practices can negatively affect the development and maintenance of clinical care (Erdmann et al., 2021; Green et al., 2023; Lee, 2009; Lee et al., 2019a; Lunshof, 2006; Ormond and Cho, 2014; Regniault et al., 2009). Tissue chips are composed of various crucial components (e.g., culture media, specialist hardware, software, etc.), each of which could, in principle, be patented or protected as trade secrets if they are not publicly disclosed (Zhang and Radisic, 2017). If other laboratories cannot access state-of-the-art research to either learn from it or improve upon older products, such restrictions can limit competition, hinder scientific innovation, and ultimately reduce the quality of tissue chip devices and therapeutics deriving from tissue chip technology.

Regulatory and research institutions should adopt measures to reduce IP barriers that hinder equitable access and innovation in tissue chip technologies. These include overly broad patent claims, restrictive licensing, and the withholding of critical data under proprietary protections (Organization for Economic Co-operation and Development, 2019). Reforms such as legally protected safe harbor provisions and publicly supported data-sharing platforms (e.g., TEX-VAL, which facilitates the exchange of validation data for MPSs) can mitigate commercial reluctance to data sharing and support greater interoperability and regulatory trust (U.S. Government Accountability Office, 2025).

### Distributive justice

Implementing tissue chip-based therapies into healthcare systems intersects with broader challenges of distributive justice, understood as the (unfair) allocation of healthcare benefits, such as access to personalized treatments, and burdens, such as exclusion from emerging therapies. It also concerns autonomy, where patients may lack the resources or support to understand or act upon complex medical information, and non-maleficence, in the potential for harm through exclusion or misuse of genetic data.

Firstly, access to tissue chip-based products varies depending on healthcare systems and the types of insurance coverage available to patients. Patients in private insurance systems or those who can afford out-of-pocket costs are more likely to access these advanced therapies, while uninsured individuals or those with limited coverage are less likely to benefit from these innovations (Brothers and Rothstein, 2015; Cornetta and Brown, 2013; Fleck, 2012, 2014, 2022; Green et al., 2023). However, with precision therapy costs averaging around €75,000 or more per patient per year, accessibility is limited even for relatively wealthy individuals (Fleck, 2022; Tabor and Goldenberg, 2018).

Secondly, when utilized in clinical care, tissue chips can support individualized disease prevention strategies. That is, medical insights provided by personalized tissue chip applications might recommend lifestyle changes that could reduce the risk of disease or mitigate its impact. However, it is argued that some patients may not have the resources or skills required to interpret complex risk information, navigate (digital) healthcare platforms, or act on medical advice (Brall and Schröder-Bäck, 2016; Brothers and Rothstein, 2015; Erdmann et al., 2021; Fusar-Poli et al., 2022; Green et al., 2023; Halley et al., 2024; Hazin et al., 2013; Iqbal et al., 2022; Knoppers and Avard, 2009; Leo et al., 2022; Newson, 2022; Shoenbill et al., 2014). As a result, the benefits of tissue chip-based therapies may be inaccessible to certain populations, potentially exacerbating existing healthcare inequities.

Thirdly, a common approach toward developing PM applications is stratification—that is, dividing patients into smaller subgroups with specific disease comorbidities to create more targeted interventions (Erikainen and Chan, 2019). It is argued, however, that dividing individuals into groups based on biological or genetic profiles could lead to discrimination and the unfair distribution of healthcare benefits (Barazzetti et al., 2021; Blumling et al., 2021; Callier, 2019; Chapman et al., 2021; Fusar-Poli et al., 2022; Gannett, 2005; Geneviève et al., 2023; Hansson, 2010; Juengst et al., 2016; Lee, 2003, 2007, 2009; Lunshof, 2006; Matthew, 2019; McClellan et al., 2013; Mensah et al., 2019; Regniault et al., 2009; Schaefer et al., 2019, 2020; Shoenbill et al., 2014; Tranvåg et al., 2021). Since PM applications have the potential to reveal subtle genetic differences and identify individuals as being at “health risk,” insurance providers or employers could use this information to discriminate against them by denying coverage or reimbursement (Brothers and Rothstein, 2015). If tissue chips are used to generate personalized health data, similar concerns about discrimination could arise. However, it is also argued that evidence for genetic discrimination remains ambiguous (Winkler and Knoppers, 2022), and its prevalence or impact in the context of tissue chips remains uncertain, too.

To support the equitable implementation of tissue chip-based innovations, policymakers and healthcare systems could establish tiered pricing models where the price of tissue chip-based diagnostics and therapeutics is adjusted based on a country’s income levels (Organization for Economic Co-operation and Development, 2018). Additionally, standardized counseling protocols could help patients understand and act on personalized test results. Finally, clear regulations such as the Genetic Information Nondiscrimination Act and the GDPR should guide the use of personalized tissue chip data to prevent discrimination or unfair treatment by insurers and employers.

## Discussion

To our knowledge, this is the first study to systematically explore the scientific literature to identify the ethical aspects of tissue chips. By integrating key ideas and concepts from the organoid, PM, and DT literature, this review provides a comprehensive overview of the ethical issues related to tissue chips in clinical and research settings. However, as tissue chips continue to evolve, new challenges may emerge. To address these challenges, ongoing reflection is essential. In what follows, we highlight several unresolved empirical and normative questions that require further research and discussion. These relate to underrepresented groups in tissue chip research, complexities and limitations of different consent models, the need for clear criteria to determine evidence standards for replacing animal models, and accountability in the standardization of tissue chip research.

Throughout the review, we engaged in multiple comparative exercises to identify gaps and ethical considerations potentially relevant to tissue chip research. Among these, the discrepancy in how the PM, DT, and organoid literature address issues of inclusivity and diversity stands out as particularly relevant to tissue chips. While PM and DT research extensively emphasize the importance of integrating diversity into datasets to improve the overall quality and accessibility of their models, these topics are significantly underrepresented in the organoid ethics literature. As mentioned in the section “evidence”, non-representative samples can produce misguided results and raise concerns about the clinical utility and scientific value of certain predictive models. This opens an important avenue for further research into diversity and inclusivity in tissue chip research. For example, what are the effects of underrepresentation in biobanks? Which forms of diversity (e.g., gender or ethnicity) should be prioritized when developing or using tissue chip models, and why? And what strategies could improve representation in biobank collections and study populations? We believe that addressing these questions is crucial for ensuring the robust and equitable scientific development of tissue chips.

A potential reason for the discrepancy mentioned here may be the overrepresentation of ethical literature on brain organoids, which predominantly focuses on related questions about sentience, consciousness, and moral status (52 of the 80 included documents specifically address these themes). While undeniably important, we contend that there are sufficient reasons why these considerations should not be overemphasized at this time (cf. Hyun et al., 2020). Specifically, brain organoids lack the complexity and connectivity observed in mature neural networks, do not receive sensory input or produce any output, and are, therefore, unable to communicate or interact with their surroundings (Koch et al., 2016; Reardon, 2018). As a result, concerns about brain organoids developing cognitive abilities such as “thinking” or achieving traits like “consciousness” or “sentience” are most likely premature. Although these limitations could in theory be overcome by applying dedicated bioengineering strategies for improving the overall maturation of brain organoids, we believe it would be beneficial for researchers and policymakers to extend their scope to also scrutinize other, more immediate ethical and practical considerations surrounding the use of organoids and other advanced *in vitro* models. A recent example of such a shift includes the collaborative bioethics approach advocated by Hyun and Lunshof (2024), which underlines the importance of interdisciplinary collaboration among scientists and non-scientists to promote the responsible innovation of brain organoid research.

Secondly, issues of informed consent, transparency, and donor autonomy are widely recognized as crucial in organoid, PM, and DT research. Yet, relatively little attention is paid to the practical challenges associated with implementing contemporary consent models. While initiatives aimed at developing consent models that are capable of addressing the complexities of contemporary research are laudable, it is relevant to further scrutinize the limitations and impracticalities of these models in real-world settings. For instance, how should researchers address the need for re-consent when donors become unreachable or pass away? Should researchers be allowed to continue to use their material, or would this breach donor autonomy? Additionally, is it realistic to suppose that re-consent documents are always written in simple language for full accessibility, particularly when simplifying the content of certain research projects might require great amounts of time and effort? Lastly, how should consent be managed when participants who donated material as minors reach adulthood, given that these individuals may have moved, their contact information is outdated, or they changed their mind with respect to donation? In light of these questions, we recommend further research into developing solutions capable of addressing these challenges.

Thirdly, while advanced *in vitro* models are often discussed in the context of the 3Rs, little attention is paid to what is truly needed to make them real alternatives to animal models. When considering how tissue chips can be positioned relative to non-human animal experimental methods, initial responses tend to focus on epistemological considerations, such as defining what constitutes reliable evidence of equivalency to animal models (Hu and Yang, 2023; Park et al., 2024; Sharoni, 2024; Wilkinson, 2019). When, for instance, is tissue chip-based evidence strong enough to justify bypassing animal models and proceeding directly to human trials? And what criteria should be used to determine whether tissue chips provide equivalent or superior predictive value compared to animal models? While these questions are highly relevant, recent work also emphasized that evidence standards do not function in isolation from the institutional practices and cultural norms that ground them (Ankeny et al., 2024; Green, 2024). This raises additional questions. What role do and should institutional structures like universities and companies play in driving (or preventing) the adoption of tissue chips in scientific practices? And in what ways do global and local disciplinary, social, and cultural norms affect the implementation of tissue chips? Understanding such factors is crucial for identifying the conditions necessary to facilitate the transition of tissue chips—and other advanced models—into mainstream scientific practices.

Fourthly, reproducibility and standardization play an important role in tissue chip research (Thakar and Fenton, 2023). Developing tissue chips involves coordinating and integrating various field-specific elements, including cells, biomaterials, engineering controls, and sensors (Leung et al., 2022; Low et al., 2021). The higher the variability between these single elements, the higher the potential for error. However, the coexistence of heterogeneous perspectives across disciplines such as chemistry, bio-computing, engineering, and biology raises uncertainties about responsibility and accountability. Indeed, standardization involves defining what constitutes reliable, reproducible, and valid tissue chip data. But if a standard proves inadequate and leads to material harm in a clinical setting, who should be held accountable? Is it the researchers who developed the model, the regulatory agencies that approved it, or the institutions that promoted its adoption? Without clearly defined roles and protocols, accountability in the field remains ambiguous, potentially undermining its credibility and adoption in research and clinical settings.

Lastly, environmental impact and sustainability receive little attention in the sampled literature, despite their importance for tissue chip development and use. Most commercially available tissue chips rely on non-biodegradable polymers, which have significant carbon footprints and limit scalable production (Core, 2023). Furthermore, tissue chip fabrication and disposal are energy intensive and their use outside laboratory settings raises concerns about waste management in contexts with limited disposal infrastructures (Ongaro et al., 2022). As the field grows, it becomes important to systematically assess, and where possible improve, the environmental footprint of tissue chips. Further work should examine which materials and fabrication processes minimize environmental impact without compromising function, how life cycle assessments can guide design, and what regulatory measures can support sustainable adaptation.

## Limitations

By examining ethical considerations across several distinct but related fields (organoids, PM, and DT), the review covered a wide range of topics. This approach provided a more comprehensive and nuanced understanding of the ethical considerations associated with introducing tissue chips in research and clinical settings than has previously been explored in the literature. There are, however, some inevitable limitations to this approach.

Firstly, mapping review methodologies are inherently broad and descriptive, which may result in a lack of depth in discussing individual topics in detail. Secondly, reviews of this kind often involve reporting bias. A different team of researchers might have chosen alternative approaches to grouping or interpreting the literature, potentially leading to a different map. Thirdly, the review was limited to English and Dutch literature, such that discussions published in other languages were not included. Lastly, the review does not incorporate recent policy documents, roadmaps, or institutional reports, which may also have limited the inclusion of ethical perspectives and discussions relevant to tissue chip research.

To address these limitations, future research could expand the review’s scope by further reflecting on specific topics as well as evaluating other relevant literature, policy documents, and reports. Future research could also adopt a case study approach that examines ethical issues within specific contexts or practices. Additionally, a comprehensive review and evaluation of policies and regulations relevant to tissue chips might further facilitate their responsible development and implementation.

## Conclusion

The goal of this review was to map the ethical issues associated with the use and implementation of tissue chips in both research and clinical settings. To achieve this, we conducted a comparative ethical analysis and identified topics across three consecutive phases—research, clinical testing, and implementation. Within these phases, nine key themes are identified: privacy and confidentiality, informed consent, evidence, ontology and moral status, animal experimentation, evidence standards, patient care, IP and commercialization, and distributive justice. By analyzing ethical considerations across the different phases of the tissue chip life cycle, we identified which issues are currently underexplored and which are well addressed within the sampled literature. This enabled us to provide several recommendations for further normative inquiry and to offer (actionable) policy recommendations where appropriate. Additionally, by addressing these issues early in the development phase of tissue chip technology, this review aims to equip policymakers with a comprehensive summary of the themes currently discussed in the academic literature—insights that can support the development of future regulations, guidelines, and governance models for the responsible use and innovation of tissue chip technology.

## Methods

A mapping review was conducted to find relevant literature across disciplinary boundaries. This methodology can be used to comprehensively survey and contextualize the existing literature on a specific topic, identify gaps, and highlight areas for further research (Grant and Booth, 2009). Given the scarcity of literature that specifically discusses tissue chip technology ethics, a comparative analytical approach was employed, expanding the inquiry to include the organoid, PM, and DT literature. These bodies of literature were included for three main reasons.

Firstly, the characteristics of organoid and tissue chip technologies share many similarities. Organoids, like tissue chips, can be grown from iPSCs and adult stem cells into various types of organ structures, including the liver, heart, and brain (Eiraku and Sasai, 2012; Nakano et al., 2012; Sato et al., 2009). As such, both utilize human biological material from donors and may offer alternatives to animal research. Moreover, some organoid and tissue chip variants exhibit neural connections and brain activity, raising questions about the potential emergence of sentience or consciousness in these models. Consequently, it is to be expected that they raise similar ethical considerations.

Secondly, tissue chips, like organoids, are used for disease modeling and drug screening and can be applied within PM approaches aimed at treating and preventing disease by considering individual biology (Green et al., 2024). PM typically involves advanced analytics applied to patient-specific biological datasets (e.g., genomic, proteomic, behavioral, self-monitoring) and covers a wide range of clinical and diagnostic strategies, including targeted therapies, pharmacogenomics, and risk stratification (Vicini et al., 2016). Organoid research intersects with PM by using patient-derived materials for pharmacogenomic screening and personalized drug testing, identifying actionable biomarkers and resistance mechanisms to guide targeted (chemo) therapies (Tao et al., 2025). Combining organoid data with broader health datasets in personalized treatment decisions raises ethical questions (e.g., around privacy, consent, and data governance) that remain largely unaddressed (Barnhart and Dierickx, 2022; de Jongh et al., 2022). We draw on the PM literature because tissue chips can raise similar questions when applied to individualized disease modeling and drug response testing that involves patient-specific data integration.

Additionally, within tissue engineering contexts, the term “DT” is increasingly used to describe (patient-specific) computational models used for predictive testing and optimization of microfluidic systems, even when these do not involve real-time data integration as in industrial DT systems (Möller and Pörtner, 2021; Portela et al., 2021; van den Berg et al., 2019). We selectively engage with DT literature where it addresses ethical questions related to model reliability, transparency, and data governance, which are central to assessing the reliability and readiness of tissue chip technologies for research and clinical applications.

To synthesize the results, we began by categorizing the findings from each body of literature into initial thematic overviews (see Tables S1–S3). These overviews were then reviewed and compared to identify recurring themes, areas of overlap, and unique, field-specific topics, as well as to exclude themes irrelevant to tissue chip contexts. A theme was deemed relevant to tissue chips if it addressed the activities or challenges that arise when using tissue chips in any of the three phases and excluded if they were not. For example, the theme *privacy and confidentiality* was included as it is directly relevant to managing sensitive personal data during the research phase. Conversely, the theme *transplantation* was excluded as tissue chips are designed for *in vitro* use and not for implantation in patients.

## Conducting a literature search

Relevant papers were identified by searching PubMed, Web of Science, JSTOR, and Philosopher’s Index using search strategies for tissue chips, organoids, PM, and DT (see Tables S5–S7). These databases were selected because they cover a broad area of biomedical (bio)ethical and philosophical manuscripts. A medical information specialist informed the choice of databases and search strategy.

## Literature selection and inclusion criteria

The following criteria were used to screen the literature (cf. Barnhart and Dierickx, 2022).

(1) The publication discusses ethical considerations directly related to the development and/or application of tissue chips, organoids, PM, or DTs in the context of biomedical research or clinical application.
(2) The ethical considerations are substantially addressed and not merely alluded to in passing.
(3) The publication is peer reviewed and appears as an academic article, book (chapter), national-level report, or working paper or as part of a PhD dissertation.
(4) The considered publication is written in either Dutch or English.

The results were collected and compiled in EndNote’s reference software, and duplicates were removed. Titles and abstracts (ti/ab) were screened by J.W., who also conducted the full-text review for final inclusion. In cases of uncertainty, J.W. discussed the selection process with N.d.G. and M.d.V. to ensure consensus.

## Data extraction, analyses, and syntheses

An initial coding frame was developed based on familiarization with the data and discussions within the research team. This frame was then expanded to include additional themes and sub-themes as they emerged during the analysis. As such, we started inspecting the literature by assigning codes to the ethical issues mentioned in each publication. Next, relevant themes and sub-themes were inferred from the assigned codes. The codes, themes, and sub-themes were formulated in an iterative process where the research team evaluated the coding frame and the themes inferred from it, a method intended to ensure that the emergent thematic structure is correct, accurately reflects the underlying data, and does not overlook any significant issues. Once themes and sub-themes were identified and verified, the precise wording used to describe them was discussed and revised when necessary.

## Quality appraisal

In line with the characteristics of mapping review methodologies as described by Grant and Booth, this approach does not include a quality assessment process (Grant and Booth, 2009). Instead, it enables a systematic collection and synthesis of the relevant literature by maintaining transparent inclusion criteria, as well as a structured data extraction and categorization, thus providing a thorough and organized overview of the field.

## Data and code availability

All data analyzed during this study were publicly available at the time of submission.

## Acknowledgments

We want to thank Tessa Phillipa, Stan Popescu, and the Walaeus Library for their guidance in designing the search strategies. We would also like to thank an anonymous reviewer for their valuable comments.

Funding: J.W. and N.d.G. are funded by reNEW, the 10.13039/501100009708Novo Nordisk Foundation Center for Stem Cell Medicine, under grant NNF21CC0073729. J.W. is also funded by the Netherlands Organ on Chip Initiative, an NWO Gravitation project funded by the Dutch Ministry of Education, Culture and Science (024.003.001).

## Author contributions

Conceptualization, J.W. and N.d.G.; methodology, J.W., M.d.V., and N.d.G.; investigation, J.W.; data curation, J.W.; writing – original draft, J.W.; writing – review & editing, J.W., M.d.V., C.M., and N.d.G.; supervision, N.d.G. and M.d.V.; funding acquisition, C.M. and M.d.V.

## Declaration of interests

The authors report there are no competing interests to declare.

## Supplemental information

Document S1. Tables S1–S7

Document S2. Article plus supplemental information
