# Supplementary material for: The ethical aspects of human organ-on-chip models: A mapping review
Source: Stem Cell Reports. 2025 Oct 30;20(11):102686. doi: 10.1016/j.stemcr.2025.102686 (PMC12790741; doi:10.1016/j.stemcr.2025.102686)
Supplement: Document S1. Tables S1–S7 [file mmc1.pdf]

Stem Cell Reports, Volume *20*

Supplemental Information

The ethical aspects of human organ-on-chip models: A mapping review

Jesse Weidema, Martine de Vries, Christine Mummery, and Nienke de Graeff

Supplemental information

Table S1. Organoids

Main theme	Sub-theme	Ethical concern	Explanation	References
Consent and commercialization	Informed consent	Autonomy and uncertainty	Rapid technological changes and unforeseen clinical applications can make it difficult for donors to understand exactly what they consent.	(Bassil and Horstkötter, 2023; Boers and Bredenoord, 2018; Boers *et al.*, 2018; Boers *et al.*, 2016; Boers *et al.*, 2019; Bollinger *et al.*, 2021; Bredenoord *et al.*, 2017; Farahany *et al.*, 2018; Harris *et al.*, 2022; Hartung *et al.*, 2023; Hyun *et al.*, 2020; Kataoka *et al.*, 2024a; Lavazza, 2019; Lavazza and Chinaia, 2023; Lensink *et al.*, 2021a; Lensink *et al.*, 2021b; Lensink *et al.*, 2020; Lewis and Holm, 2022; MacDuffie *et al.*, 2023; Mollaki, 2021; Munsie *et al.*, 2017; Ravn *et al.*, 2023; Stoeklé *et al.*, 2021)
	Commercialization and biobanking	Ownership and commodification	Donors seldom receive benefits from the commercial value of their donated materials.	(Bassil and Horstkötter, 2023; Boers and Bredenoord, 2018; Boers *et al.*, 2018; Boers *et al.*, 2016; Boers *et al.*, 2019; Bollinger *et al.*, 2021; Bredenoord *et al.*, 2017; Lavazza, 2019; Lensink *et al.*, 2021a; Lensink *et al.*, 2021b; Lensink *et al.*, 2020; Lewis and Holm, 2022; Mollaki, 2021; Ravn *et al.*, 2023; Stoeklé *et al.*, 2021)
Research and clinical application	Precision medicine	Safety, privacy and equity	Precision medicine involves collecting sensitive health data that might be misused and is extremely costly, making it inaccessible for many patients.	(Boers *et al.*, 2018; Boers *et al.*, 2016; Bredenoord *et al.*, 2017; Dam and Green, 2022; Harris *et al.*, 2022; Hyun *et al.*, 2020; Lewis and Holm, 2022)
	Transplantation	FIH; psychological impact	First-in-human clinical trials expose patients to risks; transplantation might affect a patient's bodily integrity, potentially causing emotional distress.	(Bassil and Horstkötter, 2023; Boers *et al.*, 2016; Bollinger *et al.*, 2021; Bredenoord *et al.*, 2017; Chen *et al.*, 2019; Ding *et al.*, 2022; Harris *et al.*, 2022; Hyun *et al.*, 2020; Kataoka *et al.*, 2023; Lewis and Holm, 2022; Mollaki, 2021; Munsie *et al.*, 2017; Sawai *et al.*, 2022; Schneemann *et*

				al., 2020; Stoeklé *et al.*, 2021)
Organoid ontology and moral status	Brain organoids	Consciousness and moral status	The potential to mimic neural activity and consciousness in brain organoids is vital in determining their moral status, which might force researchers to reevaluate their rights and use.	(Bayne *et al.*, 2020; Boyd and Lipshitz, 2024; Chen *et al.*, 2019; Harris *et al.*, 2022; Hostiuc *et al.*, 2019; Hyun *et al.*, 2020; Jeziorski *et al.*, 2023; Jowitt, 2023; Kataoka *et al.*, 2023; 2024a; Koplin and Gyngell, 2020; Koplin and Savulescu, 2019; Kreitmair, 2023; Lavazza, 2019; 2020; 2021a; b; Lavazza and Chinaia, 2023; 2024; Lavazza and Massimini, 2018; Lavazza and Pizzetti, 2020; Lavazza and Reichlin, 2023; Lewis and Holm, 2022; McKeown, 2023; Milford *et al.*, 2023; Montoya and Montoya, 2023; Niikawa *et al.*, 2022; Pichl *et al.*, 2023; Presley *et al.*, 2022; Sawai *et al.*, 2022; Sawai and Kataoka, 2024; Sawai *et al.*, 2019; Shepherd, 2018)
	Chimera research	Humanization and animal welfare	Introducing human biological material into animals could enhance their cognitive abilities and subjective experiences. This might affect current animal welfare regulations.	(Bassil and Horstkötter, 2023; Chen *et al.*, 2019; Ding *et al.*, 2022; Farahany *et al.*, 2018; Hartung *et al.*, 2023; Hyun *et al.*, 2020; Kataoka *et al.*, 2023; Koplin and Massie, 2021; Koplin and Gyngell, 2020; Lavazza, 2020; 2021b; Lavazza and Chinaia, 2023; Lavazza and Reichlin, 2023; Lewis and Holm, 2022; Mollaki, 2021; Munsie *et al.*, 2017; Presley *et al.*, 2022; Sample *et al.*, 2019; Sawai *et al.*, 2022; Sawai *et al.*, 2019; Stoeklé *et al.*, 2021)
	Embryo research	Classification and protection	Involves questions about gastruloids moral status and the extent to which they should be allowed to mature and experimented upon.	(Bredenoord *et al.*, 2017; Hostiuc *et al.*, 2019; Lavazza, 2021b; Lensink *et al.*, 2020; Lewis and Holm, 2022; Mollaki, 2021; Munsie and Gyngell, 2018; Munsie *et al.*, 2017; Sawai and Kataoka, 2024)
Research ethics, integrity and moral theory	Public perception and communication	Hypes and false hope	Sensational coverage of a discovery or technology can foster false hopes or unfounded worry,	(Bassil and Horstkötter, 2023; Boers and Bredenoord, 2018; Bollinger *et al.*, 2021; Bredenoord *et al.*, 2017; Chen *et al.*, 2019; Hartung

			potentially contributing to general mistrust in science.	*et al.*, 2023; Hyun *et al.*, 2020; Ide *et al.*, 2021; Iltis *et al.*, 2023; Lavazza and Chinaia, 2023; 2024; Lensink *et al.*, 2021a; Munsie *et al.*, 2017; Presley *et al.*, 2022; Ravn *et al.*, 2023; van Till and Bunnik, 2024)
	(In)justice	Unfair outcome and distribution	Unfair distribution of benefits can exacerbate existing inequalities, limiting access to advancements for underrepresented or disadvantaged populations.	(Boers and Bredenoord, 2018; Boers *et al.*, 2019; Lensink *et al.*, 2021a; Lensink *et al.*, 2020; Lewis and Holm, 2022; Mollaki, 2021; Ravn *et al.*, 2023; Sample *et al.*, 2019; Stoeklé *et al.*, 2021)

Table S2. Precision medicine

Main theme	Sub-theme	Ethical concern	Explanation	References
Confidentiality privacy and data protection	Data security and protection	Privacy	The increasing use of omics and related technologies raises concerns about data security, including risks of breaches and misuse of sensitive information.	(Adjekum *et al.*, 2017; Ahmed *et al.*, 2023; Browman *et al.*, 2014; Chow-White *et al.*, 2015; Delpierre and Kelly-Irving, 2018; Effy *et al.*, 2018; Erdmann *et al.*, 2021; Evans, 2017; Farasati Far, 2023; Fusar-Poli *et al.*, 2022; Goncharov *et al.*, 2022; Hazin *et al.*, 2013; Juengst and Van Rie, 2020; Kinkorová, 2016; Lee *et al.*, 2019a; Lewis *et al.*, 2014; Lunshof, 2006; Lysaght *et al.*, 2020; McGowan *et al.*, 2014; Ormond and Cho, 2014; Rothstein, 2021; Safarlou *et al.*, 2023; Schaefer *et al.*, 2019; Schleidgen and Marckmann, 2013; Shoaib *et al.*, 2017; Shoenbill *et al.*, 2014; Stratton and Olson, 2023; Sui *et al.*, 2023; Thapa and Camtepe, 2021; Vaszar *et al.*, 2003; Williams and Anderson, 2018; Winkler and Knoppers, 2022)
	Data sharing and integration	Interoperability and unauthorized access	As data is increasingly shared among institutions, the potential for unauthorized access grows.	(Ahmed *et al.*, 2023; Browman *et al.*, 2014; Carnevale *et al.*, 2023; Chow-White *et al.*, 2015; Egalite *et al.*, 2014; Evans, 2017; Farasati Far, 2023; Feiler *et al.*, 2017; Green *et*

				al., 2023; Hazin *et al.*, 2013; Lysaght *et al.*, 2020; Rauter *et al.*, 2021; Rothstein, 2021; Schaefer *et al.*, 2020; Sharon, 2017; Sui *et al.*, 2023; Vaszar *et al.*, 2003; Vos *et al.*, 2017)
	Informed consent	Autonomy and uncertainty	Patients must understand data use and retain autonomy over decisions, which can be challenging to ensure.	(Adjekum *et al.*, 2017; Ahmed *et al.*, 2023; Barazzetti *et al.*, 2021; Blasimme and Vayena, 2016; Browman *et al.*, 2014; Chow-White *et al.*, 2015; Effy *et al.*, 2018; Egalite *et al.*, 2014; Erdmann *et al.*, 2021; Evans, 2017; Farasati Far, 2023; Gefenas *et al.*, 2011; Goncharov *et al.*, 2022; Hansson, 2010; Kinkorová, 2016; Knoppers and Avard, 2009; Korngiebel *et al.*, 2017; Lee, 2021a; Lunshof, 2006; McGowan *et al.*, 2014; Minari *et al.*, 2018; Ormond and Cho, 2014; Parra-Calderón *et al.*, 2018; Prainsack, 2018; Regniault *et al.*, 2009; Safarlou *et al.*, 2023; Schleidgen and Marckmann, 2013; Shoenbill *et al.*, 2014; Spector-Bagdady *et al.*, 2022; Stratton and Olson, 2023; Thapa and Camtepe, 2021; Vaszar *et al.*, 2003; Vos *et al.*, 2017; Williams and Anderson, 2018; Winkler and Knoppers, 2022; Wouters *et al.*, 2021)
	Biobanking	Standardisation and financial sustainability	Standardizing sample collection and storage protocols is essential for the reliable use of biobank data, but often involves significant costs.	(Blumling *et al.*, 2021; Callier, 2019; Carnevale *et al.*, 2023; Erdmann *et al.*, 2021; Green *et al.*, 2023; Kinkorová, 2016; Lee, 2009; Lee *et al.*, 2019a; Lunshof, 2006; Ormond and Cho, 2014; Regniault *et al.*, 2009; Safarlou *et al.*, 2023)
Research ethics and integrity	Incidental findings and the communication of results	"The burden of knowledge" and right (not) to know	Patients might choose not to be informed about genetic test results for which no treatment exists.	(Ahmed *et al.*, 2023; Blasimme and Vayena, 2016; Erdmann *et al.*, 2021; Hazin *et al.*, 2013; Hummel and Braun, 2020; Ormond and Cho, 2014; Shoenbill *et al.*, 2014; Vaszar *et al.*, 2003; Williams and Anderson, 2018; Winkler and Knoppers, 2022)

	Doctor-patient relationship and genetic counselling	Resource allocation	Providing adequate counselling demands substantial staffing and recourses, which might affect the doctor-patient relationship and be infeasible for certain healthcare systems.	(Ahmed *et al.*, 2023; Blumling *et al.*, 2021; Browman *et al.*, 2014; Dion-Labrie *et al.*, 2010; Erdmann *et al.*, 2021; Gefenas *et al.*, 2011; Green *et al.*, 2023; Guadalajara *et al.*, 2022; Hazin *et al.*, 2013; Juengst *et al.*, 2016; Knoppers and Avard, 2009; Knox and Svendsen, 2023; Morley and Floridi, 2020; Myskja and Steinsbekk, 2020; Regniault *et al.*, 2009; Sabatello *et al.*, 2018; Safarlou *et al.*, 2023; Sharon, 2017; Shoaib *et al.*, 2017; Stratton and Olson, 2023; Tuteja *et al.*, 2013; Wouters *et al.*, 2021)
Health data	Data validity and medical evidence	Beneficence	A key challenge in precision medicine is determining when the available evidence is strong enough to justify introducing a new intervention into clinical practice.	(Ahmed *et al.*, 2023; Browman *et al.*, 2014; Delpierre and Kelly-Irving, 2018; Effy *et al.*, 2018; Fusar-Poli *et al.*, 2022; Gefenas *et al.*, 2011; Giusti, 2021; Green *et al.*, 2023; Hey and Barsanti-Innes, 2016; Korngiebel *et al.*, 2017; Lee, 2009; Lee, 2021b; Lewis *et al.*, 2014; McClellan *et al.*, 2013; Schleidgen and Marckmann, 2013; Shoenbill *et al.*, 2014; Vogt and Hofmann, 2022; Winkler and Knoppers, 2022)
	Big data, eHealth and AI	Sample bias	Biased data in precision medicine applications can lead to incorrect associations between health and demographics.	(Brothers and Rothstein, 2015; Carnevale *et al.*, 2023; Effy *et al.*, 2018; Hazin *et al.*, 2013; Hollister and Bonham, 2018; Hummel and Braun, 2020; Knoppers and Avard, 2009; Korngiebel *et al.*, 2017; Lee, 2021a; b; Lee *et al.*, 2019a; Morley and Floridi, 2020; Myskja and Steinsbekk, 2020; Sharon, 2017; Shoenbill *et al.*, 2014; Sui *et al.*, 2023)
	Overdiagnosis	Health surveillance and responsibilities	Medical screening programmes may increase health surveillance, imposing undue pressure and responsibility on individuals for managing their health.	(Barazzetti *et al.*, 2021; Carnevale *et al.*, 2023; Carter *et al.*, 2016; Chadwick and O'Connor, 2013; Delpierre and Kelly-Irving, 2018; Effy *et al.*, 2018; Erikainen and Chan, 2019; Farasati Far, 2023;

				Feiler, 2019; Fusar-Poli *et al.*, 2022; Gefenas *et al.*, 2011; Green *et al.*, 2023; Hollister and Bonham, 2018; Hummel and Braun, 2020; Juengst *et al.*, 2016; Juengst and McGowan, 2018; Juengst and Van Rie, 2020; Kerr *et al.*, 2018; Knox and Svendsen, 2023; Lee, 2021a; Mao *et al.*, 2024; McGonigle, 2016; Morley and Floridi, 2020; Murphy et al., 2021; Myskja and Steinsbekk, 2020; Obafemi-Ajayi *et al.*, 2022; Prainsack, 2018; Rauter *et al.*, 2021; Rothstein, 2021; Savard, 2013; Schaefer *et al.*, 2019; Sharon, 2017; Sharrer, 2017; Skantharajah *et al.*, 2023; Sui *et al.*, 2023)
	Trust	Data transparency and reliability	The complexity, size, and distributed nature of health data pose challenges for ensuring data accuracy, transparency, and reliability, which in turn can impact trust in the systems and institutions managing this information.	(Adjekum *et al.*, 2017; Ahmed *et al.*, 2023; Dion-Labrie *et al.*, 2010; Effy *et al.*, 2018; Geneviève *et al.*, 2023; Goncharov *et al.*, 2022; Green *et al.*, 2023; Hollister and Bonham, 2018; Lee, 2021a; b; Lee *et al.*, 2019a; Lee *et al.*, 2019b; Lysaght *et al.*, 2020; Minari *et al.*, 2018; Myskja and Steinsbekk, 2020; Obafemi-Ajayi *et al.*, 2022; Ong *et al.*, 2021; Rauter *et al.*, 2021; Thapa and Camtepe, 2021; Williams and Anderson, 2018)
Access and equity	High costs	Equity	Economic barriers limit patients' ability to access and benefit from personalized medicine technology.	(Brall and Schröder-Bäck, 2016; Egalite *et al.*, 2014; Erdmann *et al.*, 2021; Fleck, 2022; McClellan *et al.*, 2013; McGowan *et al.*, 2014; Ormond and Cho, 2014; Schleidgen and Marckmann, 2013; Tabor and Goldenberg, 2018; Zarif, 2022)
	Stratification and genetic discrimination	Fairness and social equity	(Racial) stratification may lead to stigmatization and discrimination against specific groups.	(Blumling *et al.*, 2021; Callier, 2019; Fusar-Poli *et al.*, 2022; Gannett, 2005; Geneviève *et al.*, 2023; Hansson, 2010; Juengst *et al.*, 2016; Lee, 2003; 2007; 2009; Lunshof, 2006; Matthew, 2019; McClellan *et al.*, 2013; Mensah *et al.*, 2019; Regniault *et al.*, 2009; Schaefer *et al.*, 2019;

				Schaefer *et al.*, 2020; Shoenbill *et al.*, 2014; Tranvåg *et al.*, 2021)
	Inclusivity and diversity	Unfair outcome and distribution	A lack of diversity in data could limit our understanding of certain pathologies or lead to the creation of products that are only accessible or effective to certain demographics	(Callier, 2019; Cohn *et al.*, 2017; Erikainen and Chan, 2019; Geneviève *et al.*, 2023; Goncharov *et al.*, 2022; Green *et al.*, 2023; Hollister and Bonham, 2018; Knoppers and Avard, 2009; Lee, 2009; Lee, 2021b; Lee *et al.*, 2019a; Lee *et al.*, 2019b; Mao *et al.*, 2024; Mensah *et al.*, 2019; Schaefer *et al.*, 2019; Schaefer *et al.*, 2020; Shemie *et al.*, 2021; Sierra-Mercado and Lázaro-Muñoz, 2018; Skantharajah *et al.*, 2023; Viaña, 2024; Vos *et al.*, 2017; Williams and Anderson, 2018; Wouters *et al.*, 2021)

Table S3. Digital twins

Main theme	Sub-theme	Ethical concern	Explanation	References
Privacy and data protection	Data security and protection	Privacy	The increasing use of omics and related technologies raises concerns about data security, including risks of breaches and misuse of sensitive information	(Bruynseels et al., 2018b; Cho and Martinez-Martin, 2023; Huang *et al.*, 2022; Iqbal *et al.*, 2022; Leo *et al.*, 2022; Popa *et al.*, 2021)
	Informed consent	Autonomy and uncertainty	Rapid technological changes and unforeseen clinical applications can make it difficult for donors to understand exactly what they consent.	(Braun, 2022; Cho and Martinez-Martin, 2023; Huang *et al.*, 2022; Iqbal *et al.*, 2022; Leo *et al.*, 2022; Tigard, 2021; Truby and Brown, 2021)
	Collection and analysis	Privacy and autonomy	The scope of data collection is often unclear, especially when service providers can collect data irrelevant for testing prespecified hypotheses.	(Braun, 2021; Braun and Krutzinna, 2022; Huang *et al.*, 2022; Popa *et al.*, 2021; Rainey, 2022)
Research ethics and integrity	Transparency and responsibility	Accountability, justice and fairness	Data-centric approaches are often opaque. Transparency in data collection and use should therefore be evaluated and discussed to reduce and account for	(Braun, 2021; Cho and Martinez-Martin, 2023; Iqbal *et al.*, 2022; Leo *et al.*, 2022; Mittelstadt, 2021; Popa *et al.*, 2021; Rainey, 2022)

			potential sources of bias.	
	Doctor-patient relationship	Patient wellbeing	Digital twins can undermine a patient's authority and autonomy in the doctor–patient relationship by providing an alternative, more "objective" account of the patient's health.	(Leo *et al.*, 2022; Mittelstadt, 2021; Popa *et al.*, 2021)
Data	Big data, AI and data first approaches	Sample bias	Digital twins are only as reliable as the data from which they are build. Missing, inaccurate or biased data might therefore distort the models, leading to unreliable predictions and false associations between health and demographics.	(Braun, 2022; Bruynseels *et al.*, 2018b; Cho and Martinez-Martin, 2023; Huang *et al.*, 2022; Iqbal *et al.*, 2022; Leo *et al.*, 2022; Mittelstadt, 2021; Popa *et al.*, 2021; Rainey, 2022)
	Models and representation, data validity and medical evidence	Technical feasibility, reliability and uncertainty	Crucial in digital twins research is determining when the available evidence is strong enough to ensure reliable predictions, as well as justify the introduction of new interventions into clinical practice.	(Braun, 2021; 2022; Braun and Krutzinna, 2022; Cho and Martinez-Martin, 2023; Loh, 2021; Popa *et al.*, 2021; Rainey, 2022)
	Wearables and eHealth	Coercion, surveillance, overdiagnosis and exclusion	The use of wearables and tracking technology could lead to the intrusive monitoring and surveillance of patients, which might coerce them into certain health practices or exclude them from certain benefits.	(Braun and Krutzinna, 2022; Bruynseels *et al.*, 2018b; Cho and Martinez-Martin, 2023; de Boer, 2020; Huang *et al.*, 2022)
Access and equity	High costs	Exclusion and inequity	High costs associated with personalized healthcare and digital twins can restrict access to only a limited few, thereby deepening healthcare disparities.	(Bruynseels *et al.*, 2018b; Cho and Martinez-Martin, 2023; Huang *et al.*, 2022; Iqbal *et al.*, 2022; Popa *et al.*, 2021)
	Digital illiteracy	Exclusion and inequity	Individuals with low digital literacy may struggle to access and benefit from	(Iqbal *et al.*, 2022; Leo *et al.*, 2022)

		personalized healthcare and digital twins, which can deepen healthcare disparities.

Table S4. Organ-on-chip search string

Data base	Search strategy
PubMed	("morals"[mesh] OR "ethic*"[tiab] OR "moral*"[tiab] OR "bioethic*"[tiab] OR "virtue*"[tiab] OR "responsibilit*"[tiab]) AND ("Microphysiological Systems"[Mesh] OR "Organ-on-a-Chip"[tiab] OR "Organ-on-a-Chip"[tiab] OR "Organ on a Chip"[tiab] OR "Organ on chip*"[tiab] OR "organ chip*"[tiab] OR "organoids on a chip"[tiab] OR "organoids-on-a-chip*"[tiab] OR "Organotypic Model*"[tiab] OR "Tissue Chip*"[tiab] OR "chip-based model* "[tiab] OR "chip based model"[tiab] OR "lab-on-a-chip"[tiab] OR "lab on a chip"[tiab] OR "lab on chip*"[tiab])
Web of Science	(WC=Ethics OR WC=Medical Ethics OR TS=ethics OR TS=morals OR (TI=ethic* OR AB=ethic*) OR (TI=moral* OR AB=moral*) OR (TI=bioethic* OR AB=bioethic*) OR (TI=virtue* OR AB=virtue*) OR (TI=responsibilit* OR AB=responsibilit*)) AND (ALL="Microphysiological Systems" OR (TI=Organ-on-a-Chip OR AB=Organ-on-a-Chip) OR (TI=Organ-on-a-Chip OR AB=Organ-on-a-Chip) OR (TI="Organ on a Chip" OR AB="Organ on a Chip") OR (TI="Organ on chip*" OR AB="Organ on chip*") OR (TI="organ chip*" OR AB="organ chip*") OR (TI="organoids on a chip:" OR AB="organoids on a chip") OR (TI=organoids-on-a-chip* OR AB=organoids-on-a-chip*) OR (TI="Organotypic Model*" OR AB="Organotypic Model*") OR (TI="Tissue Chip*" OR AB="Tissue Chip*") OR (TI="chip-based model*" OR AB="chip-based model*") OR (TI="chip based model" OR AB="chip based model") OR (TI=lab-on-a-chip OR AB=lab-on-a-chip) OR (TI="lab on a chip" OR AB="lab on a chip") OR (TI="lab on chip*" OR AB="lab on chip*"))
JSTOR	(ethic* OR moral* OR bioethic*) AND ("Organ-on-a-Chip" OR "Organ on chip" OR "organ chip*" OR "Organotypic Model*" OR "Tissue Chip*" OR "chip?based model")
Phil. Index	(ethic* OR moral* OR bioethic* OR virtue*) AND (Microphysiological Systems OR Organ-on-a-Chip OR organ chip* OR organoids on a chip OR organoids-on-a-chip* OR Organotypic Model* OR Tissue Chip* OR chip-based model* OR lab-on-a-chip OR lab on a chip OR "lab on chip*")

Table S5. Digital twins search string

Data base	Search strategy
PubMed	("Digital Twin*"[tiab]) AND ("morals"[mesh] OR "ethic*"[tiab] OR "moral*"[tiab] OR "bioethic*"[tiab] OR "virtue*"[tiab] OR "responsibilit*"[tiab])
Web of Science	(WC=Ethics OR WC=Medical Ethics OR TS=ethics OR TS=morals OR (TI=ethic* OR AB=ethic*) OR (TI=moral* OR AB=moral*) OR (TI=bioethic* OR AB=bioethic*) OR (TI=virtue* OR AB=virtue*) OR (TI=responsibilit* OR AB=responsibilit*)) AND (TS=(Digital twin) OR (TI= (digital twin) OR AB=(digital twin)) OR (TI=(digital twins) OR ab=(digital twins)))
JSTOR	("digital twins" OR "digital twin") AND ("ethic*" OR "moral*" OR "bioethic*")

Phil. Index	(Ethic* OR moral* OR bioethic* OR virtue*) AND (Digital Twin*)

Table S6. Precision medicine search string

Data base	Search strategy
PubMed	((“Precision Medicine”[MeSH] OR “precision medicine*”[tiab] OR “personalized medicine*”[tiab]) AND (“morals”[mesh] OR “ethic*”[tiab] OR “moral*”[tiab] OR “bioethic*”[tiab] OR “virtue*”[tiab] OR “responsibilit*”[tiab]))
Web of Science	((WC=Ethics OR WC=Medical Ethics OR TS=ethics OR TS=morals OR (TI=ethic* OR AB=ethic*) OR (TI=moral* OR AB=moral*) OR (TI=bioethic* OR AB=bioethic*) OR (TI=virtue* OR AB=virtue*) OR (TI=responsibilit* OR AB=responsibilit*)) AND (TS=(precision medicine) OR (TI=precision medicine* OR AB=precision medicine*) OR (TI=personalized medicine* OR AB=personalized medicine*)))
JSTOR	(“morals” OR “ethic*”) AND (“precision medicine” OR “personalized medicine” OR “individualized medicine”)
Phil. Index	(Ethic* OR moral* OR bioethic* OR virtue*) AND (Precision Medicine OR precision medicine* OR personalized medicine*)

Table S7. Organoids search string

Data base	Search strategy
PubMed	(“Organoids”[Mesh] OR “Organoid*”[tiab] OR “mini organ”[tiab:~2] OR “mini organs”[tiab:~2] OR “organ dish”[tiab:~2] OR “organs dish”[tiab:~2]) AND (“morals”[mesh] OR “ethic*”[tiab] OR “moral*”[tiab] OR “bioethic*”[tiab] OR “virtue*”[tiab] OR “responsibilit*”[tiab])
Web of Science	(TS=Organoids OR (TI=Organoid* OR AB=Organoid*) OR (TI=“mini organ” OR AB=“mini organ”) OR (TI=“mini organs” OR AB=“mini organs”) OR (TI=“organ dish” OR AB=“organ dish”) OR (TI=“organs dish” OR AB=“organs dish”)) AND (ALL=morals OR (TI=ethic* OR AB=ethic*) OR (TI=moral* OR AB=moral*) OR (TI=bioethic* OR AB=bioethic*) OR (TI=virtue* OR AB=virtue*) OR (TI=responsibilit* OR AB=responsibilit*))
JSTOR	(“organoid*” OR “mini organ*” OR “organ dish”) AND (“ethic*” OR “moral*” OR “bioethic*”)
Phil. Index	(Ethic* OR moral* OR bioethic* OR virtue*) AND (“organoids” OR “organoid*” OR “mini organ” OR “mini organs” OR "organ dish” OR “organs dish”)
